# A139 SUBMUCOSAL TUNNELING ENDOSCOPIC RESECTION (STER) FOR ESOPHAGEAL GIST: A CASE REPORT

**DOI:** 10.1093/jcag/gwae059.139

**Published:** 2025-02-10

**Authors:** A DASHTI, S X Jiang, R Winter, R Trasolini

**Affiliations:** Gastroenterology, The University of British Columbia Faculty of Medicine, Vancouver, BC, Canada; The University of British Columbia Faculty of Medicine, Vancouver, BC, Canada; Gastroenterology, The University of British Columbia Faculty of Medicine, Vancouver, BC, Canada; Gastroenterology, The University of British Columbia Faculty of Medicine, Vancouver, BC, Canada

## Abstract

**Background:**

Gastrointestinal stromal tumors (GISTs) are rare mesenchymal tumors found throughout the gastrointestinal tract, with esophageal GISTs being particularly uncommon. Surgical resection is the standard treatment option, though it is associated with significant morbidity. Submucosal Tunneling Endoscopic Resection (STER) is a minimally invasive technique for per-oral subepithelial tumor removal with preservation of the surrounding esophagus and no need for thoracoscopy or mediastinoscopy. Herein, we report a successful case of STER for esophageal GIST in an elderly Canadian patient.

**Aims:**

To present the successful management of an esophageal GIST using submucosal tunneling endoscopic resection.

**Methods:**

Case report and literature review.

**Results: Case Report:**

An 80-year-old man with dysphagia underwent an EUS for a 1.5 cm subepithelial lesion identified in his mid-esophagus on EGD. EUS showed a hypoechoic lesion arising from the muscularis propria. Biopsy confirmed a spindle cell tumor, C-KIT positive consistent with GIST. The patient was offered transthoracic surgical resection but declined given the associated risks and complications. An interval CT scan demonstrated a 4 mm increase in the size of the tumor. The patient was referred for consideration of endoscopic resection. He ultimately underwent submucosal tunneled endoscopic resection of the GIST with no complications. The procedure was performed by creating a mucosal flap over the GIST via submucosal tunnelling with subsequent traction assisted full thickness myotomy surrounding the lesion while preserving the capsule, followed by clip closure of the mucosotomy. Pathology confirmed R0 resection with low mitotic rate. A routine esophagram post-procedure showed no contrast leakage, and the patient was discharged the following day.

**Literature Review:**

STER is a minimally invasive technique for resection of gastrointestinal subepithelial lesions first reported in 2012. It offers several advantages including preservation of GI tract function, rapid recovery time, and low adverse event rate but requires careful lesion selection and technical facility with endoscopic submucosal dissection, myotomy and defect closure. Studies have demonstrated high rates of complete resection with low complication risks, particularly in treating small to medium-sized tumors. To our knowledge, this case represents the first reported use of STER in Canada.

**Conclusions: Conclusions:**

STER is a minimally invasive option for resection of subepithelial lesions. In this case it was used effectively with readily available equipment in a Canadian hospital. It reduces recovery time, preserves esophageal structure, and minimizes complications.

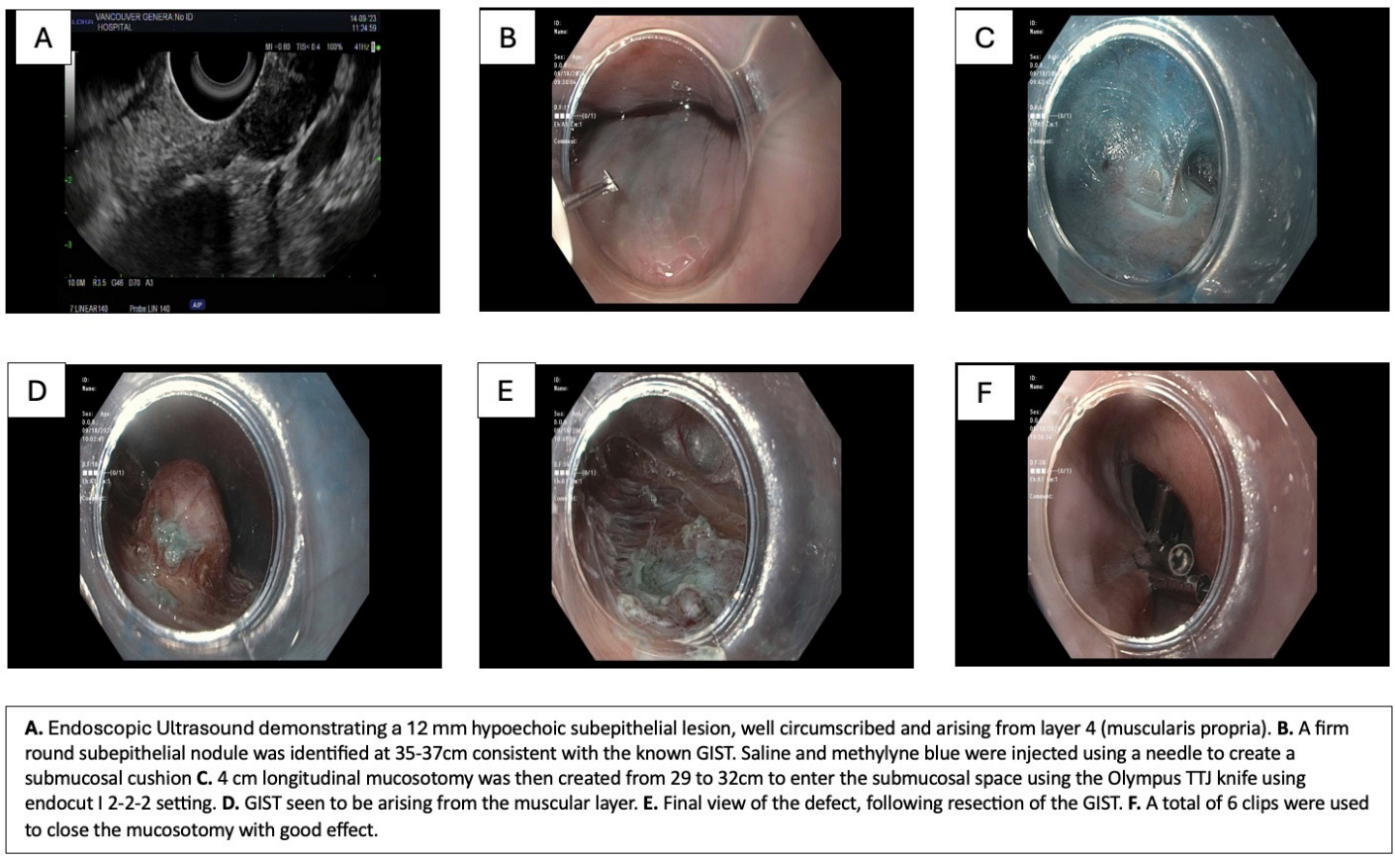

**Funding Agencies:**

None

